# Happiness Is in the Field

**DOI:** 10.3390/ani15213087

**Published:** 2025-10-24

**Authors:** Dominique-Marie Votion

**Affiliations:** Department of Functional Sciences, Faculty of Veterinary Medicine, Pharmacology and Toxicology, Fundamental and Applied Research for Animals & Health (FARAH), University of Liège, 4000 Liège, Belgium; dominique.votion@uliege.be

## Editorial

Common sense suggests that ‘happiness is in the field’, as grasslands are considered environments that promote the health and well-being of grazing animals. However, they can present toxicological risks that are sometimes poorly known, so there is certainly a need to update our knowledge of pasture-associated poisoning.

This Special Issue gathers articles that explore both well-documented and emerging threats to livestock and horses linked to their grazing environment.

The collection includes case reports and investigations on acute poisonings caused by well-known toxic plants such as oak (*Quercus* spp.), where ingestion of large quantities of acorns can lead to death in both cattle [1] and horses [2,3]. Another study highlights the role of cyanogenic plants like sorghum, in which environmental stressors such as drought may exacerbate the production of toxins like dhurrin, triggering cyanide poisoning in cattle [4].

One of the most emblematic examples of a growing pasture-associated disease is equine atypical myopathy [5]. This condition, linked to ingestion of protoxins from seeds or seedlings of the sycamore maple (*Acer pseudoplatanus*), has emerged over the last two decades as a major cause of death in pastured horses across Europe. Although *A. pseudoplatanus* is considered an indigenous tree, its role in pasture toxicity was only recognised a decade ago [6]. Moreover, this syndrome is no longer restricted to horses as *A. pseudoplatanus poisoning* has been confirmed in other species [7,8,9,10]. This illustrates that pasture poisoning is not a static phenomenon: it is shaped by changing ecosystems, grazing behaviour [11], and climate changes. Insights into the equine faecal microbiota in the context of atypical myopathy suggest that gut microbial activity may modulate toxin metabolism and susceptibility [12]. Understanding these host–toxin interactions could open up new approaches to prevention.

Environmental toxicology also encompasses chronic and subclinical exposures. One study in this issue assesses the concentrations of toxic and essential trace elements in cow’s milk in north-eastern Brazil, showing that proximity to major roads correlates with high levels of lead in milk, a potential public health problem [13]. Such findings remind us that contamination does not always present as acute disease but can emerge insidiously through bioaccumulation and long-term exposure.

Throughout history, toxic threats have evolved, from classic cases involving heavy metals [14,15] to current concerns such as pesticides [16] and per- and polyfluoroalkyl substances (PFAS) [17]. Effective management of pasture-associated poisoning requires both robust diagnostic tools [18] and a mechanistic understanding of toxic processes [19], enabling not just treatment but prevention [20].

As the welfare of grazing animals is intimately linked to pasture [21,22,23], we need to ensure that pasture remains a safe environment, whether for leisure animals or for livestock in food production systems. This Special Issue reminds us of the necessity to be aware of the risks associated with pasture to strengthen our ability to protect animal health in an ever-changing environment.
